# Aiding eco-labelling process and its implementation: Environmental Impact Assessment Methodology to define Product Category Rules for canned anchovies

**DOI:** 10.1016/j.mex.2017.03.001

**Published:** 2017-03-22

**Authors:** Jara Laso, María Margallo, Pére Fullana, Alba Bala, Cristina Gazulla, Ángel Irabien, Rubén Aldaco

**Affiliations:** a Departamento de Ingenierías Química y Biomolecular, Universidad de Cantabria, Avda. de los Castros, s.n, 39005 Santander, Spain; b UNESCO Chair in Life Cycle and Climate Change, Escola Superior de Comerç Internacional (ESCI-UPF), Pg. Pujades 1, 08003 Barcelona, Spain; c Lavola Cosostenibilidad Rbla, Catalunya, 6, 08007, Spain

**Keywords:** Product Category Rules (PCR) of canned anchovy based on Environmental Sustainability Assessment (ESA) method, life cycle assessment, canning industry, environmental product declaration, product category rules

## Abstract

To be able to fulfil high market expectations for a number of practical applications, Environmental Product Declarations (EPDs) have to meet and comply with specific and strict methodological prerequisites. These expectations include the possibility to add up Life Cycle Assessment (LCA)-based information in the supply chain and to compare different EPDs. To achieve this goal, common and harmonized calculation rules have to be established, the so-called Product Category Rules (PCRs), which set the overall LCA calculation rules to create EPDs.

This document provides PCRs for the assessment of the environmental performance of canned anchovies in Cantabria Region based on an Environmental Sustainability Assessment (ESA) method. This method uses two main variables: the natural resources sustainability (NRS) and the environmental burdens sustainability (EBS). To reduce the complexity of ESA and facilitate the decision-making process, all variables are normalized and weighted to obtain two global dimensionless indexes: resource consumption (X_1_) and environmental burdens (X_2_).

• This paper sets the PCRs adapted to the Cantabrian canned anchovies.
• ESA method facilitates the product comparison and the decision-making process.
• This paper stablishes all the steps that an EPD should include within the PCRs of Cantabrian canned anchovies.

## Graphical abstract

**Figure:**
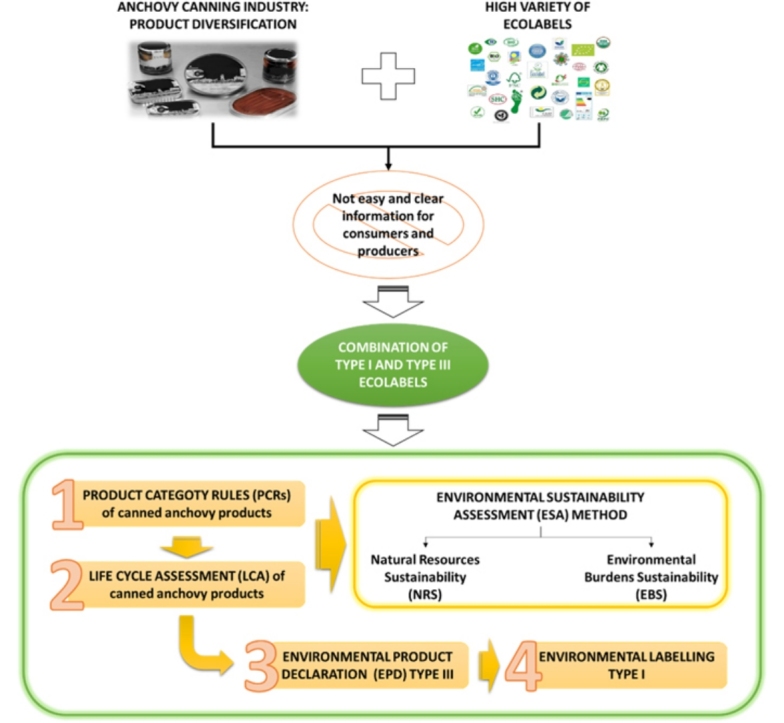

**Specifications Table**

**Table:** 

Subject area	Select one of the following subject areas: • Chemical Engineering
More specific subject area	*Describe narrower subject area*
Method name	*Product Category Rules (PCR) of canned anchovy based on Environmental Sustainability Assessment (ESA) method*
Name and reference of original method	*Product category rules (PCRs) according to ISO 14025:2006. Product group: UN CPC 2124. Fish, otherwise prepared or preserved; caviar and caviar substitutes.*
Resource availability	Gabi Software

## Method details

To perform the Life Cycle Assessment (LCA) of the individual products it is necessary to define the Product Category Rules (PCRs). PCRs document is defined in ISO 14025 [1] as a set of specific rules, requirements and guidelines for developing Type III environmental declarations for one or more product categories. This PCR document specifies the rules for the underlying LCA [2], [3] a sets minimum requirements on EPDs for a specific product group.

In particular, this work defines the PCRs for the canned anchovy products of Cantabria Region (North of Spain). This industry has a high product diversification due to the great worldwide competitiveness and demand, which makes necessary the development of marketing strategies to reach and maintain a leading position in the market. The canned anchovy sector has developed a wide range of new products using several types of oil; and packaging; and anchovy species [4]. In previous works, authors have evaluated the management of the anchovy residues generated during the canning process [5] and have assessed the environmental performance of the production of one can of anchovies in olive oil (from gate to grave), proposing several improvements to reduce its environmental impact [6].

Up to date, the only EPD programme which has published a PCR document for canned fish is the *International EPD^®^ System* [7] throughout the document *“Fish, otherwise prepared or preserved; caviar and caviar substitutes”* [8]. However, there is any developed EPDs using this PCR. This work is based on this PCR document proposing a new impact assessment method. Usually PCRs apply CML 2001 [9], a set of metrics that in some cases could be difficult to understand for producers and consumers and thus confuse the process comparisons. The proposed method, named Environmental Sustainability Assessment (ESA) reduces the complexity of LCA improving the comprehension of the results and thus assist the decision-making process [10]. In Section PCRs of canned anchovy products, the ESA method is explained and then, in Section Life cycle inventory analysis, the PCRs of canned anchovy products are described.

## Method: environmental sustainability assessment (ESA)

The ESA method includes the 4 steps proposed in the ISO 14040 [2], [3]: classification, characterization, normalization and weighting.

### Classification and characterization

Classification includes the selection of the impact categories, and the characterization models of the study. In the characterization stage the impact of each emission or resource consumption is modelled quantitatively using a characterization factor or potency factor (PF). This factor expresses how much that flow contributes to the impact category indicator. The ESA methodology is based on two main variables: the natural resources sustainability (NRS) (i.e., depletion/exhaustion) and the environmental burdens sustainability (EBS) (i.e., air, water and land).

In this way, NRS includes the consumption of final useful resources such as energy (*X*_1,1_) [MJ], materials $\left(X_{1,2}\right)$ [kg] and water$\left(X_{1,3}\right)$ [kg]; thus, it can be described by a NRS dimensionless *X*_1_ index

On the other hand, EBS includes the primary burdens to the air, water and land due to the release of pollutants (i.e., gas, liquid and solid state). EBS is given by the environmental sustainability metrics developed by IChemE [11] and is classified in 12 variables grouped into the release to each environmental compartment (i.e., air, water and land).

This set of indicators can be used to measure the environmental sustainability performance of an operating unit, providing a balanced view of the environmental impact of inputs (i.e., resource usage), and outputs (i.e., emissions, effluents, and waste).

### Normalization and weighting

Normalization relates the magnitude of impacts in different impact categories to reference values. The aim of normalization is two-folded: (a) to place the life cycle impact assessment (LCIA) indicator results into a broader context; and (b) to adjust the results to have common dimensions. This procedure allows the decision maker to track the progress towards environmental sustainability and to clarify the optimization procedure, at least for the environmental pillar.

Normalization facilitates the comparison among impact categories, while weighting procedure ranks the different environmental categories according to their relative importance [12]. Accordingly, NRS an EBS metrics were normalized and weighted to assess the contribution of each variable within a single index.

In this way, internal normalization is applied to the NRS and EBS impact categories according to Eqs. (1)–(2). The average consumption of natural resources ${\bar{\text{X}}}_{1,i}^{\text{ref}}$ and the average environmental impacts ${\bar{\text{X}}}_{2,\text{j},\text{k}}^{\text{ref}}$) for the scenarios under study are used as references, respectively.

(1) $${\text{X}}_{1,\text{i}}^{*}=\frac{{\text{X}}_{1,\text{i}}}{{\bar{\text{X}}}_{1,\text{i}}^{\text{ref}}}$$

(2) $${\text{X}}_{2,\text{j},\text{k}}^{*}=\frac{{\text{X}}_{2,\text{j},\text{k}}}{{\bar{\text{X}}}_{2,\text{j},\text{k}}^{\text{ref}}}$$

where *i* represents different NRS (energy, materials and water); *j* represents air and water compartments; *k* represents the environmental impact categories; X_1,i_ is the consumption of each *i* NRS; ${\text{X}}_{1,\text{i}}^{*}$ is the normalized value of X_1,i_; ${\bar{\text{X}}}_{1,\text{i}}^{\text{ref}}$ is the NRS reference value; X_2,j,k_ describes the *k* environmental impact category for the environmental compartment *j*; ${\bar{\text{X}}}_{2,\text{j},\text{k}}^{\text{ref}}$ is the reference value for each impact category and ${\text{X}}_{2,\text{j},\text{k}}^{*}$ is the normalized value of X_2,j,k_.

The ${\text{X}}_{2,\text{j}}^{*}$macro-categories are obtained by subjecting the normalised impact categories to equal weighting (Eq. (3)). This approach is based on the assumption that the same relevance is attributed to each impact category and, thus, equal relative weights are given. Since there are 5 EBS impact categories to each environmental burden, 1/5 is assumed as weighting factor (*δ_j,k_*). Despite being not science-based, this weighting scheme is usually applied in the literature as a first approximation for constructing a composite index [13].

(3) $${\text{X}}_{2,\text{j}}^{*}=\sum_{\text{j}=1}^{j}\delta_{j,k}\cdot {\text{X}}_{2,\text{j},\text{k}}^{*}$$

Where *δ_j,k_* represents the weighting factor for each *k* impact category in each *j* environmental compartment. ${\text{X}}_{2,\text{j}}^{*}$ represents the macro-categories EBS to air and EBS to water.

Therefore, the normalized NRS macro-categories$({\text{X}}_{1,\text{i}}^{*}$) that represent the consumption of energy (${\text{X}}_{1,1}^{*}$), materials (${\text{X}}_{1,2}^{*}$) and water (${\text{X}}_{1,3}^{*}$) and the normalized EBS macro-categories (${\text{X}}_{2,\text{j},}^{*}$) that describe the impact to air (${\text{X}}_{2,1}^{*}$), water (${\text{X}}_{2,2}^{*}$) and land$({\text{X}}_{2,3}^{*}$), can be grouped into a single index so-called Environmental Sustainability Index (ESI). The weighting procedure proposed is shown in Eq. (4).

(4) $$ESI=\gamma_{1}\overset{}{\underset{}{\sum_{i=1}^{i}}}\alpha_{\text{i}}{\text{X}}_{1,\text{i}}^{*}+\gamma_{2}\sum_{i=1}^{i}\beta_{\text{j}}{\text{X}}_{2,\text{j}}^{*}\text{i}\epsilon \left[1,3\right]\text{and}\text{j}\epsilon \left[1,3\right]$$

Where ESI is the global environmental sustainability index that combines the consumption of NRS and the generation of EBS. The weighting factor α_i_ serves at the aggregation of the 3 NRS macro-categories (${\text{X}}_{1,\text{i}}^{*}$) into a single super-category namely NRS (X_1_). Similarly, β_j_ enables the weighting and combination of the 3 EBS macro-categories (${\text{X}}_{2,\text{j}}^{*}$) into EBS (X_2_) index. Finally, *γ_i_* combines X_1_ and X_2_ super-categories into the composite ESI index.

Most of the composite indexes are constructed by equal weighting [14]. Hence, for comparison issues, the three NRS and the three EBS macro-categories are first assumed to be equally relevant. However, using the same weighting value for α_i_ and β_j_ may result in an imbalance structure within the composite index, since the dimension grouping the larger number of variables (${\text{X}}_{1,\text{i}}^{*}$) will have higher weight than the dimension resulting from the aggregation of lesser variables (${\text{X}}_{2,\text{j}}^{*}$). For this reason, Eqs. (5)-(6) must be satisfied and thus α_i_ = 1/3 for each *i* and β_j_ = 1/3 for each *j*.

(5) $$s.t.\sum_{\text{i}=1}^{\text{i}=\text{n}}\alpha_{\text{i}}=1$$

(6) $$\sum_{\text{j}=1}^{\text{j}=\text{n}}\beta_{\text{j}}=1$$

This methodology will help the decision maker choose the best option, reducing its complexity because the two main functions can be converted into comparable variables that can be used later in a multi-objective optimization.

## PCRs of canned anchovy products

### Functional unit definition

The functional unit (FU) is 100 g of edible product plus the packaging weight (primary and secondary). Covering liquids or preservatives are considered edible, their weight is intended to contribute to the declared unit (i.e. refined olive oil, extra virgin olive oil or sunflower oil). Primary packaging varies from aluminium can, tinplate and glass jar within a cardboard box, whereas secondary packaging shall be composed by a carton box and LDPE film for the transportation.

### System boundaries

The procedure is separated into three different life cycle stages (see Fig. 2) and the environmental performance associated with each of the three life cycle stages shall be reported separately.

#### Upstream processes (from cradle-to-gate)

a) Anchovy fishing

**Fig. 1:**
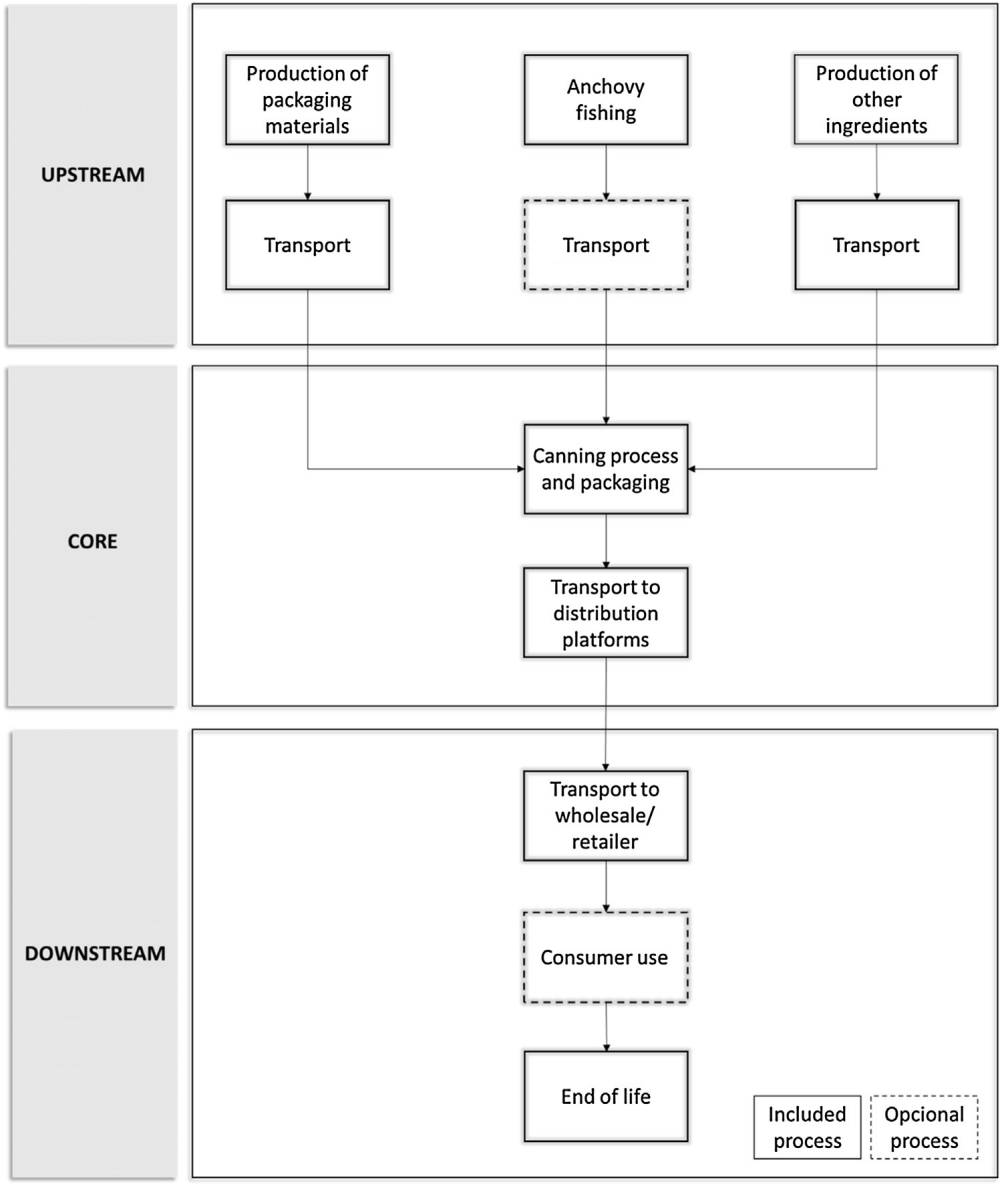
System diagram illustrating the main processes and the division into upstream, core and downstream processes.

**Fig. 2:**
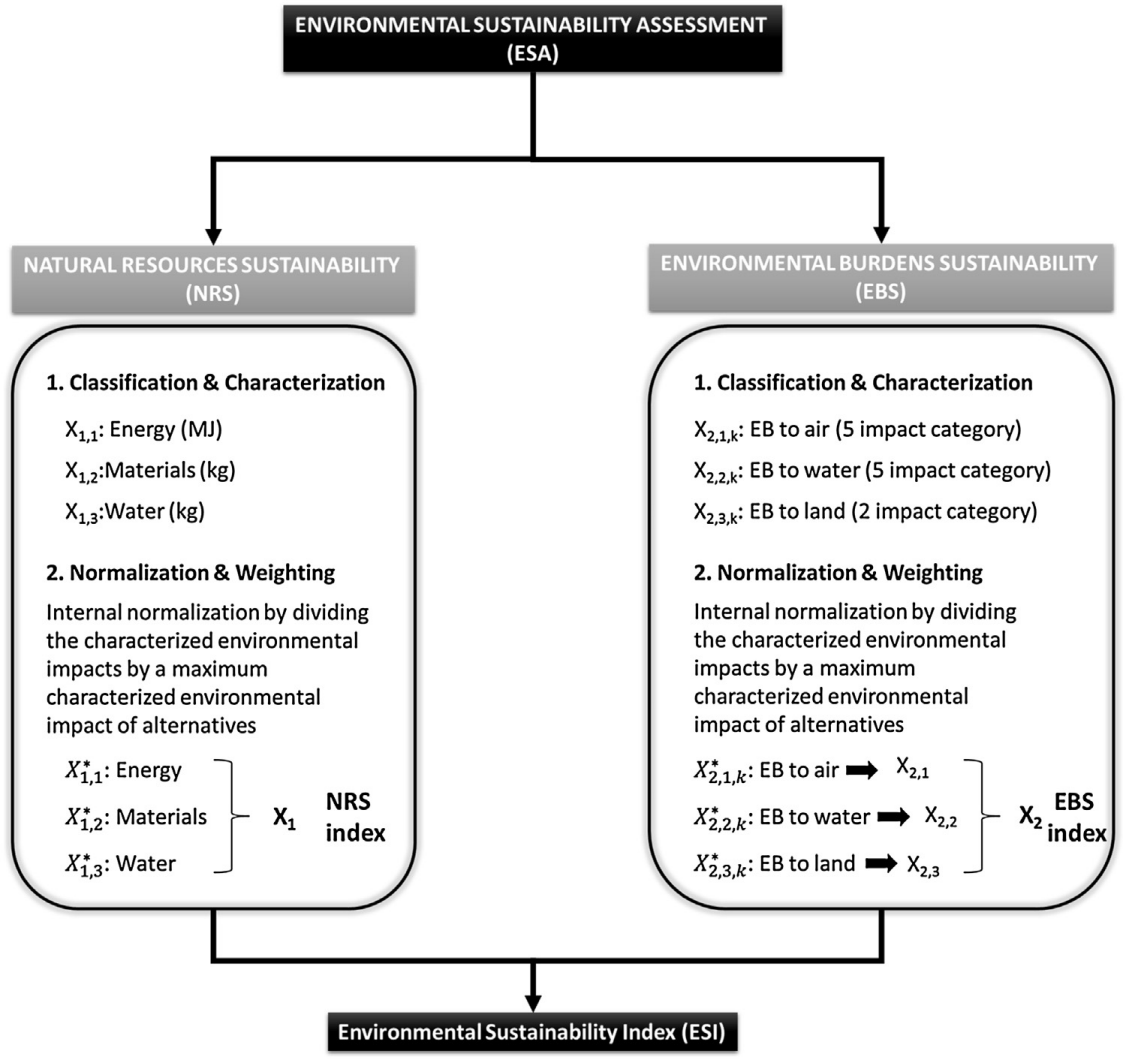
Environmental Sustainability Assessment (ESA) method.

The stages of the vesseĺs life cycle shall be encompass: construction (hull, engines and fishing nets), maintenance (hull, engines, fishing nets, antifouling and boat paint), use (diesel, ice, lubricant oil, residues), and end of life (vessel dismantling) (Fig. 1).

For each of these process, it shall be considered the flows of energy and materials, as well as the residues and emissions generated.

b) Production of other ingredients

This section shall include the production of secondary ingredients: salt, brine and oil (refined olive oil, extra virgin olive oil, and sunflower oil).

For each of these processes, it shall be considered all flows of energy and materials needed.

c) Production of packaging materials

The production of the primary packaging (aluminium can, tinplate, glass jar and cardboard box) and secondary packaging (carton box and LDPE film) shall be depicted.

For each of these processes, it shall be considered all flows of energy and materials needed.

d) Transport

If the anchovy origin is Argentina, Chile or Peru, the transportation of the anchovy by cargo ship to Cantabria Region shall be considered.

The transportation of the fresh anchovy from the harbor to the canning plants by van shall only be considered if the distance is larger than 5 km.

The transportation of the other ingredients and packaging shall be carry out by road by truck considering the real distance covered by the ingredients and packaging.

#### Core processes (from gate-to-gate)

a) Canning process and packaging

This module shall collect all the processes in the canning plant, from the fresh anchovy reception to the packaging and storage of the canned anchovy. The following processes shall be considered: pre-salting, removal of heads, curing, scalding, cutting, filleting, canning, oil filling, closing of cans, washing of cans, primary packaging, and secondary packaging and storage.

For each of these processes, it shall be encompassed the auxiliary products and materials (water, cleaning detergent, laboratory material, etc.), as well as the energy consumption and the residues generated.

b) Transport to distribution platforms

The transportation of the canned anchovy products to a distribution platform by truck shall be considered if it is relevant.

#### Downstream processes (from gate-to-grave)

a) Transport to wholesale/retailer

The distribution scenario shall be defined and declared into the EPD. When applicable, data have to provide the following information:

• Average transport scenarios inside the production country.
• Average transport scenarios from the production country to the country where the products are consumed.
• Average transport scenarios from the product country to the continents where the products are consumed.

Canned anchovies are a semi-preserved product, therefore, the energy needed for the refrigeration in the wholesale/retailer shall be included.

b) Consumer use

Consumer use of the canned anchovy shall only be considered if the canned anchovies are stored in household freezers.

Regarding the consumption patter, canned anchovies are ready-to-eat products and they do not require any cooking.

The main assumptions made for the use phase shall be declared in the EPD.

c) End of life

The management of the residues generated from the consumption of canned anchovies (aluminium can, tinplate, glass jar and cardboard box) shall be detailed in the EPD considering a management scenario representative of the region.

Recommendations for the recycling of packaging materials and the potential environmental impact and benefit of recycling shall be given.

### Cut-off rules

Life cycle inventory (LCI) data for a minimum of 99% of total inflows to the core module shall be included. Inflows not included in the LCA shall be documented in the EPD. The summary of the excluded inflows cannot exceed 5% of the total energy and materials used in the whole life cycle of the canned anchovy product.

The construction, production and maintenance of necessary infrastructures and equipment shall be excluded of EPD based on the long estimated lifespan.

### Allocation rules

Allocation rules must be defined for individual products when the manufacturing processes result in many different kinds of products and where there is only aggregate information available about the total level of emissions. Collection of product-specific information under this circumstances is to prefer to avoid allocation, by dividing the unit process into two or more sub-processes and collecting the environmental data related to these sub-processes or by expanding the system boundaries.

If allocation cannot be avoided, the priorities suggested by the ISO 14040 shall be considered in the procedure definition. In this case, the inputs should be allocated between the products and functions based on physical relationships (i.e. mass). If physical relationships cannot be established or used, allocation can be based on other relationships, for example economical allocation. Any other allocation procedures must be justified.

## Life cycle inventory analysis

### Data quality

Primary data shall be used for the core module. These specific data are gathered from the actual manufacturing plant(s) where specific processes are carried out. Secondary data from other parts of the life cycle traced to the specific product system under study (i.e. secondary ingredients, packaging materials or electricity) can be provided by a contracted supplier or by Ecoinvent^®^ [12] and PE [13] databases.

The LCI data shall be representative for the time period for which the EPD is valid (maximum three years). Data collected should be referred to a time period of one year, except the anchovy fishery stage where it is encouraged the use of three consecutive years, at least, to minimize the effect of the stock abundance.

The data for the core module shall be representative for the actual production processes and representative for the site/region where the respective process is taking place.

For the electricity used in the process, if the company buys the energy from the electricity mix on the actual market, the national electricity mix shall be adopted and the electricity mix used shall be documented.

### Units and quantities

The International System of Units (SI units) shall be used. Reasonable multiples may be adopted for a better understanding. A maximum of three significant digits shall be used when reporting LCA results.

## Environmental impact assessment

**Fig. 3:**
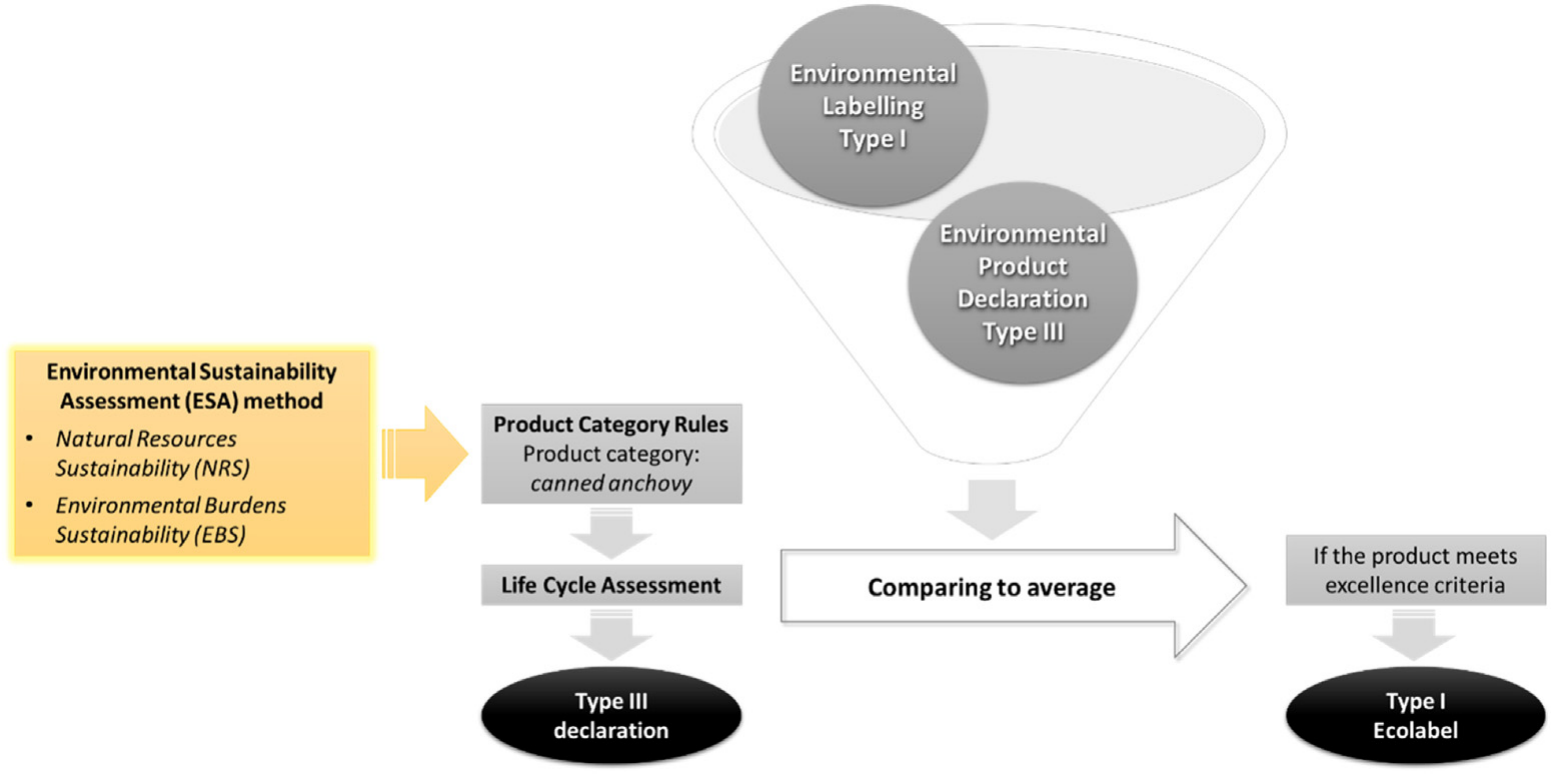
Combination of Type I and Type III eco-labels using the Environmental Sustainability Assessment (ESA) method to define the Product Category Rules (PCRs) of canned anchovy products.

The environmental impact per functional unit for the following environmental impact categories shall be reported in the EPD, divided into core, upstream and downstream module. The environmental method used shall be the ESA method defined in Section PCRs of canned anchovy products. Fig. 3 shows a scheme of the ESA method.

## Content of the EPD^®^

• Specification of the manufacturing company
• Specification of the product and the functional unit considered
• Description of the main product processes according to the system boundaries
• Life cycle inventory data
• Environmental performance-related information
  ○ Use of resources
  ○ Potential environmental impacts

• Information about any life cycle stage omitted
• Other relevant information

## Additional information

Product ecolabeling

Today there is an increasing interest across Europe in establishing guidelines that facilitate and drive the measurement and communication of the environmental behavior of products and organizations [15]. Ecolabels are used by manufactures and distributors to provide information about the environmental performance of their good on a voluntary basis. When accurate and relevant, this information should help consumers to identify those products and services of the market with lower environmental impacts. The ISO 1402X family of standards [16] proposes three types of eco-labels as possible environmental communication instruments that can be applied by companies:

• Environmental Labelling (Type I): a voluntary program that awards a license to authorize the use of environmental labels on products indicating overall environmental preferability of a product within a particular product category based on life cycle considerations [17].
• Self-declared environmental claims (Type II): informative environmental self-declaration claims [18].
• Environmental Declarations (Type III): voluntary programs that provide quantified environmental data of a product, under pre-set categories of parameters set by a qualified third party and based on life cycle assessment, and verify by that or another qualified third party [1]. From the consumer´s point of view, some studies show that little is known about the meaning of ecolabels, and interpreting the environmental information offered by them is often quite confusing. Only 7% of consumers believe that ecolabels provide sufficient, clear and easy to understand information about the environmental impact of products, whereas 32% think that ecolabels provide sufficient information, but that it is not altogether clear [19].

Within this context, Environmental Product Declarations (EPDs) (Type III) seem to be the ideal tools to prevent this confusion from arising among consumers and to make environmental comparisons between products/services easier. This is due to the fact that they represent a set of environmental indicators based on the application of the Life Cycle Assessment (LCA) methodology to the product/service under study following specific pre-defined calculation rules (named Product Category Rules, PCR). However, the technical and detailed contents of EPDs make them better suited for professional purchasers rather than final consumers, which may have the time and competence to understand their contents. On the other hand, Environmental Labelling (Type I) is easier to understand, however the extent in which LCA methodology is followed in the definition of awarding criteria varies from one Type I scheme to another. The use of Self-declared environmental claims (Type II) are not recommended in this case because they are developed by manufacturers, no third-party verified, and they do not use LCA since they are usually based in best practices. This work proposes a combination of Type I and Type III ecolabels to reach both final consumers and sector experts. This ecolabel offers both detailed quantitative information and visual information to identify the most environmentally friendly product. Producers first develop an EPD (Type III) of their products following the PCRs and then, by comparison to average market reference values of the different environmental impact categories, companies award an Ecolabel Type I for their products if they satisfy the threshold values.

This scheme (see Fig. 3) implies that Ecolabel Type I criteria should be based on LCA results of individual products.
